# Dynamic Ecosystem Adaptation through Allostasis (DEA-A) Model: Conceptual Presentation of an Integrative Theoretical Framework for Global Health Change

**DOI:** 10.3390/ijerph21040432

**Published:** 2024-04-02

**Authors:** Guillaume Broc, Lionel Brunel, Olivier Lareyre

**Affiliations:** EPSYLON EA 4556, Paul Valéry Montpellier 3, University of Montpellier, 34090 Montpellier, France; lionel.brunel@univ-montp3.fr (L.B.); olivier.lareyre@univ-montp3.fr (O.L.)

**Keywords:** theoretical framework, model, allostasis, adaptation, global health, ecosystem

## Abstract

Achieving ambitious goals in Global Health first requires an integrative understanding of how individuals and organizations adapt in a living ecosystem. The absence of a unified framework limits the consideration of the issues in their complexity, which further complicates the planning of Global Health programs aimed at articulating population-based prevention and individual-level (clinical) interventions. The aim of the conceptual contribution is to propose such a model. It introduces the Dynamic Ecosystem of Adaptation through Allostasis (DEA-A) theoretical framework, emphasizing the functional adaptation of individuals and organizations in symbiosis with their living ecosystem. The DEA-A framework articulates two central components to grasp the complexity of adaptation: the internal dynamics (intrasystem level) and the environmental dynamics (ecosystem level). It bridges diverse conceptual approaches, including stress and adaptation models, behavior-change models, and ecosystem-based perspectives. Epistemological considerations raised in the conceptual article prompt a reconsideration of methods and tools for the planning of intervention. Further contributions will present a suitable methodology for the application of the DEA-A framework along with practical recommendations.

## 1. Background

The systemic impact of changes on the health of individuals, human organizations, and ecosystems has been underscored by the World Health Organization [WHO] in a recent report (particularly through the interconnected nature of pandemics, climate, and economic, political, and social crises) [1]. Aligned with these challenges, concepts have emerged that better account for the complexity of health processes. The concepts of One Health and EcoHealth thus emphasize the link between human health and animal health, one from an immunology perspective (risk of infectious transmissions), and the other from a socio-economic and environmental perspective (impact of biodiversity on socio-economic ecosystems) [2,3]. Global Health and Planetary Health are other concepts that emphasize the importance of understanding health on a global scale [2]. According to this definition, health, well-being, and equity within human civilization are dependent upon a sustainable and nature-respecting economic and social policy that acknowledges its dependence on natural systems. It is proposed that these definitions be incorporated under the term ‘Global Health [GH]’ in order to provide greater transversality [4]. The term clearly conveys the challenge of adopting 1—a comprehensive perspective on the determinants or issues directly or indirectly affecting health; 2—an interdisciplinary and multidisciplinary approach to health, both within and beyond the Health Sciences spectrum (for example, by integrating the Humanities and Social Sciences); 3—interventional strategies that better align with the complexity of the problem, encompassing both individual- (e.g., clinical) and population-based approaches, or targeting the ecosystem as a whole.

The promotion of GH would thus require a thorough understanding of both the functioning and adaptation processes, not only at the intrasystem level of actors (people, policy makers, healthcare providers, etc.) and human organizations (e.g., healthcare systems, businesses, and government) [A/O] but also at the ecosystem level of the environment capable of impacting and being impacted by these adaptations, and that is itself constantly evolving. It is, however, not a simple matter to provide a concrete response to these ambitions. As a matter of fact, the current research has demonstrated the benefits of integrating theoretical aspects into the planning of complex programs [5,6,7,8]. In addition to defining the rationale for the program (i.e., content and mechanisms of action), providing theoretical anchoring would be an asset for better understanding the context of the intervention and anticipating deployment issues (such as appropriation and implementation factors). There are a plethora of theories and models that have been developed to account for the biopsychosocial adaptation of individuals and behavioral changes [9,10,11,12]. According to Michie et al. [13,14], most of them cover only a small subset of relevant constructs or overlap considerably. Therefore, a transversal reading of these contributions is necessary in order to capture conceptually and in its complexity the process of adaptation of individuals and organizations within their living ecosystem.

Due to its transtheoretical potential, the homeostasis paradigm is an ideal framework for exploring a system’s adaptive process in a broad sense. Homeostasis is enshrined in Natural Science but has been extended since to the psychology of human adaptation, emotional self-regulation, and behavior-process explanation [15,16,17,18,19,20,21]. Several authors have suggested that homeostasis can be applied to entire societies [22]. And, it may even represent a universal phenomenon [23,24], since it occurs at multiple levels of systems (from cells to living organisms, societies, ecosystems, and even the entire planet). This would be, therefore, an essential paradigm when it comes to capturing the adaptation process of individuals and organizations within a living ecosystem in a GH approach. A Dynamic Ecosystem Adaptation through Allostasis [DEA-A] model has, thus, been formulated, which is based on the principles of homeostasis.

## 2. Objective

This conceptual article aims to provide a theoretical introduction to the Dynamic Ecosystem Adaptation through Allostasis [DEA-A] model, as well as a conceptual reflection to help provide a better understanding in a GH approach of how actors and organizations function and adapt within a dynamic and complex ecosystem.

## 3. The Dynamic Ecosystem Adaptation through Allostasis [DEA-A] Framework

The DEA-A model is based on a substantial theoretical foundation in Natural Sciences and Human and Social Sciences, especially in Psychology (notably in health psychology, social and organizational psychology, and cognitive psychology) and in Public Health. It suggests that adaptation can take different forms but proceeds from the same functions. The model DEA-A describes the adaptation process at two levels. The first concerns the intrasystem level of actors and organizations [A/O] and accounts for their internal functioning. It will be presented in the first part of the article. The second, addressed in a second section, refers to the ecosystem level and accounts for functioning in context, which considers mutual influences of the A/Os’ adaptation and the characteristics of the surrounding environment.

### 3.1. Intrasystem Level of Adaptation of A/Os in the DEA-A Model

**Figure 1:**
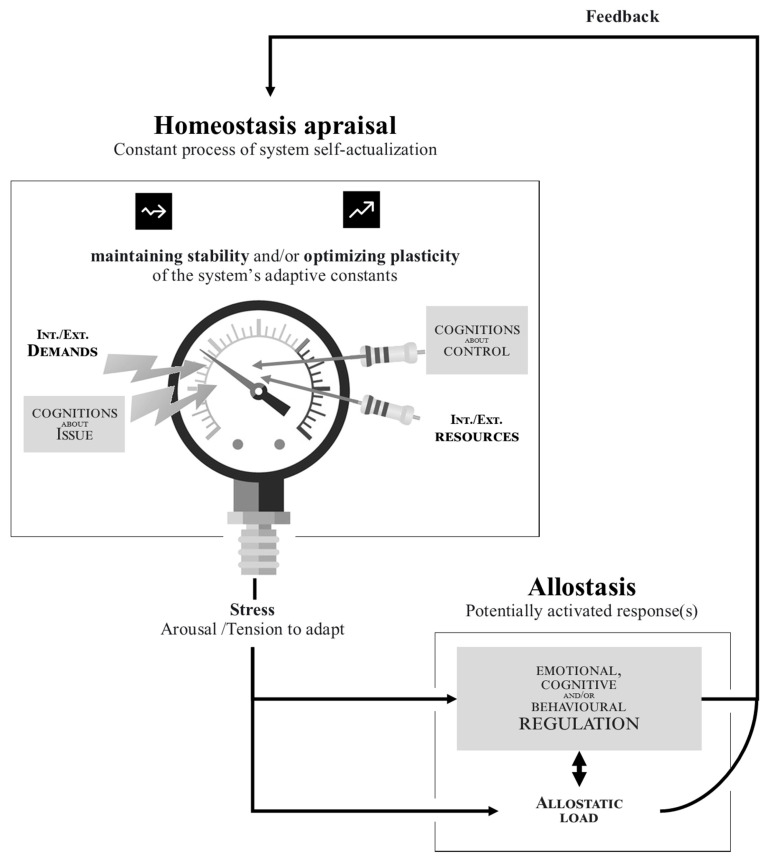
Adaptation process at the internal (structural) level of a system according to a Dynamic Ecosystem Adaptation through Allostasis [DEA-A] model. Notes. Homeostatic constants are the resources enabling the satisfaction of needs documented in well-being theories (i.e., competence, relatedness, autonomy). As the homeostasis/allostasis [H/A] process is cyclical, the same resource (like health) can both determine and be determined by the H/A process. In the same way, allostatic responses determine future homeostatic states and appraisals as well as subsequent regulations. The *H/A process is mainly unconscious.* Elaboration likelihood/awareness depends on 1—the level of the pressure (effect size) and the system’s resources/conditions to detect it (power); 2—cognitive efforts to infer and trace the process from arousal or identified allostatic responses. The *H/A process is mainly automatic*. Control over the H/A process is possible when i—elaboration likelihood is high; ii—past allostasis allows it (e.g., CR such as planning or hypervigilance); iii—certain resources (e.g., executive functions: inhibition capacities and cognitive flexibility) allow this.

Figure 1 illustrates the homeostasis/allostasis adaptation process at the internal level of A/Os in the DEA-A model. According to the model, stress emerges when the functioning of the A/O is challenged, resulting in behavioral, emotional, and/or cognitive reactions (allostatic regulations) aimed at restoring homeostasis. The key component in the Figure will be the subject of a description in the following sections, supplemented by Table 1, which sheds light on the theoretical constructs and the determinants (not exhaustive) likely to be mobilized in the DEA-A model.

#### 3.1.1. Homeostasis and Adaptation Constants

Homeostasis refers to the ability to maintain a system’s internal balance [25]. The nature and definition of the constants, i.e., the parameters of this equilibrium that serve as indicators of the overall health of a system, differ according to the observed unit (e.g., survival criteria differ depending on whether the system is an individual, an organization, or two species of animals that are phylogenetically distant). Adaptation is achieved for an individual when they are able to maintain a relative balance across their biological, psychological, and social dimensions [19,21,26]. Homeostasis is closely related to humanistic concepts such as actualization and self-actualization, which reflect the innate tendency of organisms to explore and develop their capacities to maintain and enhance their states and, for humans, to utilize their potential to improve their lives [27,28]. A primary objective of homeostasis is to maintain system stability (by preserving the A/O’s needs, as described elsewhere [27,29,30]) and, only then, to ensure that it grows (by acquiring new resources and/or achieving higher-level needs) in order to enhance its plasticity (i.e., its resilience capability) in the face of environmental contingencies. When balance is challenged, the system becomes motivated to adapt by triggering an allostatic response, otherwise known as “Allostasis” [31,32].

#### 3.1.2. Homeostasis Appraisal

As a system’s internal and external environments continuously change, its relative balance can be affected or disrupted. In fulfilling a particular need, A/Os may be objectively confronted with the alteration of a fundamental resource (object, condition, norm, value, support, energy, etc.) [29,33]. Additionally, they may be prone to subjectively evaluating the stakes pertaining to that loss, and anticipating further threats based on cognitive processes. Transactional theories of stress [34,35], and, by extension, emotional regulation [36,37], and self-regulation theories [38,39,40,41,42], have now well-documented these processes of primary (perceived stress) and secondary appraisal (perceived control). The former concerns the assessment of the stake posed by the situation (threat to stability and challenge/opportunity to enhance plasticity), while the latter focuses on the state of resources to cope with this stake. The demands from the internal and external environment, along with the cognitive evaluation of the stakes, exert an adaptive pressure, whereas the individual’s resources and the cognitive evaluation of control exert a resistance force against such a pressure. From a neurocognitive perspective, the Free-Energy Principle (FEP) suggests that biological systems, including the brain, strive to minimize a quantity known as free energy, which is a measure of the imbalance between the system’s internal model of the world and its sensory inputs [43]. According to this theory, systems evolve to minimize the free energy of their internal models, thus promoting adaptive behavior and efficient information processing. A stress response is triggered when demand exceeds resources or when a high level of free energy is reached, which serves as a signal to motivate regulation. The evaluation of homeostasis is not necessarily a conscious process, nor even a cognitive appraisal (e.g., it may only be physiological). Furthermore, emotions are bound to interfere, as we will see later on. As a result, people tend to focus on short-term contingencies (short-term threats and opportunities), particularly when the biopsychosocial situation (which entails the financial situation) is too unstable for them to plan for the future or invest in limited resources [41,44,45]. Cognitive resources such as executive functions enable the individual to project and prioritize more distal contingencies [38,41,46,47,48].

#### 3.1.3. Stress and Needs for Regulation

In physiology, stress is an alarm signal (i.e., tension) that prepares the organism for self-defense and/or expansion [31,49]. Stress triggers allostasis, which allows homeostasis to be restored. The stress remains adaptive, as long as the level of arousal remains well tolerated by the system, and a change is promptly provided to terminate the signal (see allostatic load below). The nomological network of stress includes constructs such as physiological reactions (i.e., hunger, thirst, fatigue, and excitement), emotions (i.e., fear, sadness, disgust, anger, surprise, joy, and boredom), and normative discomforts (i.e., cognitive dissonance and guilt) [36,50,51]. The forms of stress are specific to a given homeostatic imbalance, informing and guiding the system to achieve balance. It broadly represents the idea of ‘drive’, ‘need for regulation’, and even ‘motivation’, which could manifest in different ways depending on whether it is referring to an individual or a larger entity such as an organization system. For example, one might refer to an institution (hospital, school) that is in crisis, ‘under tension’, or ‘with needs in...’. It is also important to point out that arousal can differ in intensity as well as duration, which can affect allostatic activity (e.g., low levels of stress may not motivate, while excessive tension may paralyze) [49,52,53,54]. Signals can occur concurrently, but the ones activated last gradually erase/scramble the older signals. Individuals differ subjectively when it comes to their susceptibility to stress, and there is also the phenomenon of stress desensitization as well [55,56,57,58]. Due to the feedback-loop process, stress itself can confer internal pressure and, when conditions and resources allow for it, may also be cognitively appraised and elaborated/rebuilt (see further).

#### 3.1.4. Allostatic Response

Henri Laborit defined three modes of stress response in human ethology [59]: 1—flight (a preferred reaction since it keeps the stressor and its consequences at a distance); 2—fight (which requires more resources and is more risky); 3—resignation (a palliative strategy for conserving and restoring resources, which can be useful when waiting for an opportunity to cope differently, but which results in significant allostatic load). Having identified the three possible directions of system regulation (‘move away’ or ‘move towards’ or ‘stand still’), these modes provide individuals with a wide range of adaptation strategies, many of which are well documented, notably by theories such as coping [34,60], emotion regulation [36,37], self-regulation [39,61], and defense mechanisms [62,63]. Three types of allostasis can be distinguished. First, emotional regulation [ER] targets the arousal directly, by attempting to reduce it (e.g., biofeedback, relaxation), or to become accustomed to it (e.g., frustration tolerance/endurance, and desensitization). Cognitive regulation [CR], on the other hand, targets the reappraisal of the situation so that stakes and stress can be eliminated (e.g., distorting the perceived threat by minimizing risk, and/or perceived control by overestimating one’s resources, rationalizing how important it is to seize a certain opportunity, etc.). Finally, behavioral regulation [BR] targets, through verbal or non-verbal actions, a concrete modification of the internal environment (e.g., taking anti-fatigue medication) or external environment (e.g., limiting one’s activities) likely to change the balance of power between the demands of the situation and the resources necessary to withstand them, thereby removing the stake. It is possible for both allostatic reactions to occur simultaneously [Note. *One or more allostatic responses may be produced for a given signal, without taking into account the porosity between the regulations. For instance, alcohol consumption is a BR since it modifies the internal environment, shunting thoughts and overloading the system with other emotions (e.g., joy, anger) or anesthetizing them, constituting then forms of CR/ER*]. In addition, automatisms exist in the use of either of these regulatory pathways (i.e., the procedural nature of the response), especially in situations with familiar parameters [41]. Responses that are directly accessible are activated automatically, although control can be exerted (through planning) over the prioritization and selection of responses [Note. *The DEA-A model does not conceptualize intention other than as the outcome of causal attribution, i.e., a cognitive reconstruction a posteriori made by the individual based on what he perceives of his functioning (i.e., BR, CR, and ER) in a given context. Planning, on the other hand, is considered as part of the homeostasis/allostasis process. This high-level CR is activated when automatic allostasis fails to relieve the stress whose allostatic load increasingly weighs on the system. Planning is dependent on a number of internal resources (e.g., executive functions, self-regulation capacities) as well as external resources (e.g., opportunities for action, professional support for decision-making). The A/Os exercise control over the regulation in this instance. They prioritize requests experienced as pressing and response options perceived as having a favorable proximal cost–benefit ratio, taking into account the resources which have to be allocated or invested]*. Functioning habits are favored since their outcome has already been experienced, which makes their further activating more certain and less energy-consuming (they do not require a continuous activation of resources) [64,65,66]. Hence, allostatic responses that are perceived as effective (not necessarily functional), in the short term, without any contingent constraints or risks (i.e., that the individual feels confident handling) are preferred and then likely re-adopted.

#### 3.1.5. Feedback, Reappraisal, and Reinforcements

Reinforcement is, indeed, a well-documented construct in behavioral [67,68], neurocognitive [69], and social–cognitive [70] theories of learning. Allostatic responses that are integrated by the system as successfully protecting, restoring, or consolidating the expression of needs are positively reinforced, while those that have no effect or are detrimental are not reinforced or negatively reinforced. Allostasis responses that have no recognized adaptive function by the system are prone to extinction over time. This feedback is influenced by both personal experiences and those of others (i.e., vicarious experiences). According to the neurocognitive approach of FEP, the repetition or disappearance of a provided regulation does not depend on the value of the stimulus but rather is a means of minimizing surprise hence free energy within the system by adopting an optimal response [71]. A key point to consider is that, due to these learnings and the fact that the system periodically makes micro-adjustments in response to constant pressures from the internal and external environments, returning to equilibrium never amounts to returning to the pre-adjustment state. It is particularly true given that the regulation in question has been able to modify environmental parameters. In such cases, allostatic responses can be the source of new imbalances (i.e., residual tensions); for example, when satisfying a specific need (e.g., relatedness: ‘sharing a cigarette among friends’) compromises the integrity of another (e.g., competence: ‘concern about lung cancer’/autonomy: ‘addictive attitude to smoking and submission to the judgment of others’). The ability to recognize this process still exists and will depend, again, on the cognitive resources allocated to the evaluation (i.e., the mobilization of high-level versus low-level operations) [72]. There are potential errors in interpretation (i.e., identification) as well as attribution, both of which may influence the reappraisal of homeostasis [73,74,75].

#### 3.1.6. Allostatic Load as the Cost of Adaptation

Beyond the detrimental effects of the changes in both the internal and external environment, the global wear and tear inherent in the process of allostasis results in a weakening of the system so long as the need for regulation endures. The cost of adaptation is referred to as ‘Allostatic Load’ [31,32,76,77]. It can be viewed as the degradation of a system as a result of stress, which manifests as a depletion of internal resources mobilized to deal with the stakes. Allostatic load increases as 1—the arousal is intense, tetanizing the person’s regulatory capacity. 2—The allostatic state persists, such as when adaptive responses fail or new discomforts occur, which are too frequent and/or with too short a latency to allow the system to recover. 3—The activity of adaptive systems is either excessive (hyperactivity or ‘overstress’) or too episodic (hypoactivity or ‘understress’), leading in both cases to a dysregulation of allostasis. As part of allostatic load, we refer not only to a physiological erosion of the organism caused by anarchic cortisol release but also to non-exhaustive constructs such as inflammatory responses, impaired cognitive performance, difficulty regulating emotion, or decision-making tension [77,78,79,80,81]. It is important to consider such an impact of stress on the entire adaptation process (which is cyclic, as depicted in Figure 1). By depleting resources, allostatic load interferes with both the allostatic response (by making costly strategies less likely, as well as those with uncertain or distal returns on investment) and homeostasis evaluation (for example, by allocating fewer cognitive resources to the processing of environmental information, thereby favoring evaluative biases [72,73]).

The DEA-A model is illustrated in Box 1 to account for the intrasystem functioning of A/Os in GH.

Box 1 **Illustration of the DEA-A model to account for the functioning at the intrasystem level of A/Os in GH.**

As an illustration, the healthcare system obeys constants that govern its homeostasis and must be monitored. The question is generally whether a system can meet its needs for competence (for example here, to ensure its care missions), relatedness (e.g., to have a meaningful place in society and a recognized role), and autonomy (e.g., to operate freely and without interference, for example, from lobbies) and evaluate it as such. If there is no crisis, the system can invest new resources to secure this adaptation and grow (i.e., reinforce its plasticity). Otherwise, demands (e.g., an increase in admissions, an increase in resistance pathologies, and pandemics) can exceed resources (carers, equipment, endowments, and establishments), impact the constants (i.e., challenge stability), and put the healthcare system under tension. Stress has several manifestations that are all indicators of the health of the system (e.g., team turnover, caregiver exhaustion, deteriorating quality of care, increase in adverse events, and medical wandering) and further deplete the system’s resources.

Among the Global Health [GH] priorities supported by the WHO is the reduction of health inequalities among people with disabilities. Achieving such an objective requires consideration and intervention at several individual and structural levels (e.g., education, healthcare, employment, political, and social systems). The intrasystem level of the DEA-A concerns the analysis of the specific functioning of each of the systems (e.g., the healthcare system as a whole), as well as each individual actor (e.g., the attending physician), category of actors (general practitioners), organizations (such as a given healthcare facility), or even categories of organizations (such as the pharmaceutical industry) within this system, depending on their contribution to the GH issue.

Based on the DEA-A framework, analyzing how the A/O, in this case the healthcare system, self-regulates stress and seeks balance through allostasis is proposed, whether it is focused on emotion (e.g., replacement of carers without addressing turnover; simple archiving of annual reports), on cognition (e.g., rationalization: ensuring hospital beds vs. quality of care; depersonalization of patients; trivialization of the problem) and/or on behavior (such as requesting subsidies, reorganizing services, or arranging consultation circuits/organization of care). The intrasystem functioning must be captured for at least two reasons. First, it is likely to represent an (allostasic) load for the A/O (the healthcare system) and/or not to be functional anymore over time (e.g., the reorganization of care can generate stress among caregivers and worsen the situation in the long run). Second, it can contribute to GH problems (e.g., medical wandering of persons with disabilities, longer admissions time, discrimination, and institutional violence). This intrasystem functional analysis within the DEA-A framework allows for a better understanding of the situation of each A/O and categories of A/Os, as well as consideration of initial avenues for intervention and their effects.

### 3.2. Ecosystem Level of Adaptation of A/Os in the DEA-A Model

**Figure 2:**
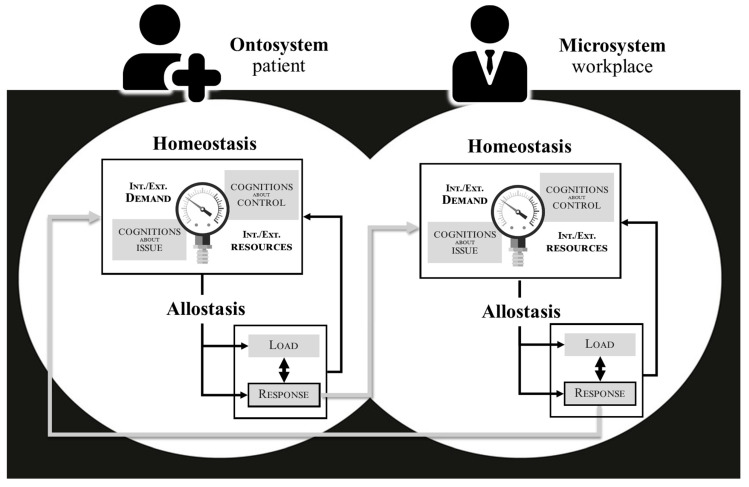
Illustration of the inter-influence in the adaptation of two systems in the DEA-A model. Notes: According to the figure, a patient’s self-regulation (e.g., extending sick leave) can influence the specific homeostasis of the workplace microsystem (e.g., threatening to fail to meet deadlines and tarnishing the company’s image), even a specific actor’s balance within it (for instance, the repercussions of an employee’s sick leave on the subsequent mental load and physical fatigue of colleagues). In turn, these impacted entities are likely to produce allostatic responses that may modify their internal and external environments, leading ultimately to a direct or indirect effect on the patient (e.g., implicit pressure to return to work, exclusion, or displacement).

According to ecological models of human development and behavior [82,83,84], organisms are far from isolated [85]. Rather, they are immersed in and interconnected within a broader living ecological niche [Note. *The concept refers to “a set formed by the environment (i.e., specific characteristics of a particular context, e.g., those of the patient) and by a community of individuals who evolve within this environment and modify it (e.g., actors from various systems—family, social circle, primary and specialized healthcare, business, public health policies—interact with each other and with the individual)”*] that permeates their evolution and trajectories over time. Such a perspective corresponds to the vision of the organism as an autopoietic system, i.e., referring to the dynamic process of self-maintaining and the self-reproducing nature of living systems, enacting and, thus, arising from the ongoing, reciprocal interactions between an organism and its environment [86]. Figure 2 already provides insight into how, at a dyadic level, two A/Os may mutually affect each other in their intrinsic functioning in the DEA-A model.

**Figure 3:**
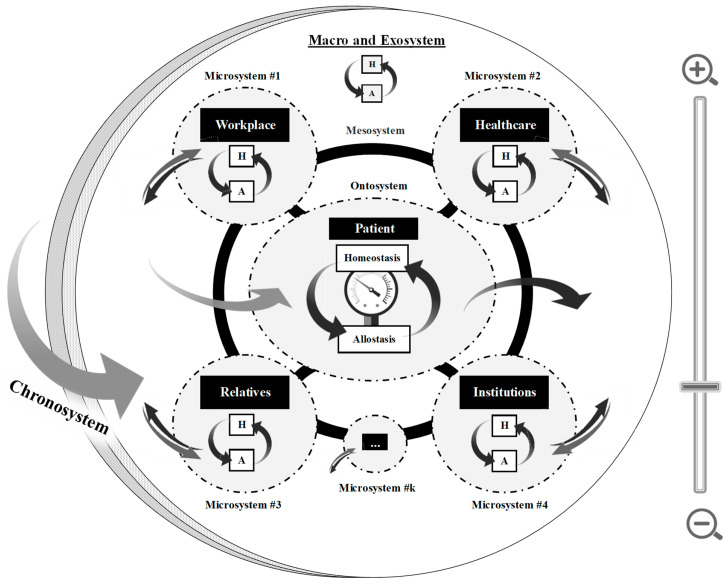
The Dynamic Ecosystem Adaptation through Allostasis [DEA-A] framework emphasizing how individuals and organizations adapt in a living ecosystem. Notes: Every system, from the most microscopic (e.g., cells) to the most macroscale (e.g., society, planet), is governed by the homeostasis/allostasis process. The Figure illustrates both the pressures faced by a given system and the resources that it consumes/exploits from another system and vice versa. The reciprocal influence between a system and its ecosystem is symbolized by 1—the thick black connections between the systems represented, i.e., between the ontosystem and the microsystems, and between the microsystems (i.e., the mesosystem). 2—The solid gray and black arrows between these systems and the exo-/macrosystems. Zoom bar represents the ability to go down to a more microscopic level, and thus see subsystems within a larger system (e.g., the manager, the executives, the employee, the union representative, etc. within the workplace system). Also, it allows for zooming out to go to more macrolevel levels (e.g., geopolitical). Mentions #1–4 evoke the different possible microsystems for a given ontosystem. Not all are represented (hence the ‘#k’). The actors/organizations considered and their assigned ontosystem/microsystem position vary depending on the focus and the situation under study.

The entire representation of the DEA-A model is provided in Figure 3, which illustrates more broadly the homeostasis/allostasis adaptation process at the ecosystem level of A/Os. According to the DEA-A model, several nested systems contribute to the adaptation of A/Os within their environment, which encompass 1—the intrasystem dynamics of each A/Os’ adaptation governed by the homeostasis/allostasis process (ontosystem; see previous section); 2—the relationship dynamics with (microsystem) and between A/Os (mesosystem); 3—the influence of context, whether political, legal, or institutional (exosystem), or even cultural, representational and societal (macrosystem); and 4—the temporal dynamic indicative of the minor and major evolutions that affect the niche over time (chronosystem).

By emphasizing ecosystem, we first and foremost acknowledge that each system, and the actors within it, regulate their own adaptation needs without attempting to ensure that other systems are kept in equilibrium [Note. *The case of whistleblowers is quite illustrative. Employees who become aware of institutional mistreatment or food contamination with E. coli bacteria may alert the institution’s hierarchy (institutionalized allostatic response), which will then configure a stress signal for the organization. In response, the institution could self-regulate through emotional (e.g., disregarding alerts), cognitive (e.g., minimizing the situation), and/or behavioral (e.g., establishing new directives) allostatic responses. There is, then, a possibility that it may recover its own equilibrium, without necessarily and sustainably resolving the issue. This situation can lead to employees collectively stopping the activity (going on strike) or alerting the public to seek allostasis from an overall system (e.g., the state, the justice system). Whistleblowers may, therefore, result in additional stress for the institution, which could result in their actions being criticized or made detrimental for them. It is, therefore, the most shared functioning of employees to maintain the activities under threat of losing their jobs and jeopardizing their individual personal/family balance. As a result, they become inclined to more cognitive (e.g., dilution of responsibility, external justifications) and emotional means of regulation (e.g., sensitization, an increase in tolerance thresholds). Even though socially understandable or even acceptable, this instinctive register of adaptation adversely impacts other A/Os, such as families who may face loss or vulnerability. As a result of this lack of symbiosis in the adaptation of an A/O, whistleblower protection measures are employed to ensure that the ecosystem operates properly*]. By extension, the same regulation (e.g., adoption of telecommuting) could be adopted by two systems, but serve different adaptive purposes (e.g., competence for the company: ‘maintaining activity’ vs. relatedness for the employee: ‘more time with the family’). It is, however, important to seek a functional adaptation between systems (or symbiosis) at the risk of an escalation of regulation and that chronic homeostatic imbalances may eventually disrupt the balance of the surrounding systems or the entire ecosystem (i.e., butterfly effect). Since environmental resources (e.g., social support for patients) are internal resources for others (e.g., time, attention, and emotional investment for caregivers), their exploitation can alter the balance within both systems (e.g., increased patient quality of life versus burden for caregiver, thereby deteriorating the relationship). In this way, the allostatic load paradigm should be extended from the intrasystem level of the A/Os to the relational dynamics between them (e.g., resistance as a degradation of communication, relationship, or therapeutic alliance between two stakeholders), up to the entire ecosystem (e.g., decline/erosion of resources until rupture occurs). In Figure 3, the double arrows illustrate this porosity of systems and their imprint on the environment, i.e., between what each consumes as resources and produces as a result of its own adaptation (new requirements, constraints, and/or resources). The developments are reflected in the chronosystem. It should be noted that temporality is not necessarily objective or quantifiable and that each system evolves at its own pace.

The DEA-A model is illustrated in Box 2 to account for the functioning at the ecosystem level of A/Os in GH.

Box 2 **Illustration of the DEA-A model to account for the functioning at the ecosystem level of A/Os in GH.**

Concerning the Global Health [GH] issue relating to health inequalities affecting people with disabilities, the ecosystem functional analysis guided by the DEA-A framework aims to place in context the functioning of actors and organizations [A/Os] (here, businesses, social partners, healthcare providers, local authorities, transport operators, etc.). The ontosystem reflects the intrasystem functioning of an A/O or a category of A/Os (see previous section). The microsystem reflects the interrelationship of an A/O with other A/Os, whereas the mesosystem reflects the interrelationship of at least two microsystems indirectly affecting the ontosystem. As the focus of the analysis changes, the ontosystem does not exclusively embody the target population (e.g., people with a disability), but rather all of the A/Os (e.g., the workplace can sometimes be the microsystem of the target audience, and sometimes the ontosystem when its own functioning is examined in the situation). A conceptualization of this nature in the DEA-A model is not trivial and seeks to illustrate how each A/O has its own goals and adaptation priorities, as well as being a stakeholder in the solution as such. Based upon the DEA-A framework, one can analyze to what extent the functioning of two A/Os can co-exist, synergize (symbiosis), or can be even detrimental to one and/or the other (e.g., hospitalization and treatment under constraint denying people’s autonomy; companies’ reluctance to provide employees with disabilities with reasonable accommodations, resulting in accessibility issues or workplace accidents). This means that the allostasis of some represents the demands, constraints, or resources of others. In addition, determinants can be identified within the exosystem (e.g., regulations concerning the obligation to employ workers with disabilities or policies that emphasize individual compensation rather than transitioning to a more inclusive society) and macrosystem (e.g., societal values, stereotypes about mental disability) that influence the process of homeostasis/allostasis [H/A] of A/Os. The systems (politics, society) are themselves governed by an H/A process. At the ecosystem level, the DEA-A framework facilitates the setting of change objectives by A/O in order to achieve symbiosis in their allostasis. The complexity of GH issues calls for a refocusing of the analysis on the smaller scale of a country or territory.

## 4. Discussion

This conceptual article presents an overview of the Dynamic Ecosystem Adaptation through Allostasis [DEA-A] model, a framework designed to provide a theoretical anchor in Global Health enabling a comprehensive understanding of A/Os’ functioning and adaptation within a complex living ecosystem. Two central components were considered by the DEA-A model: 1—the internal dynamics (intrasystem level), which conceives of the adaptation processes mobilized by individuals—and by extension the actors (e.g., the medical advisor) or organizations/systems (as a unit) of the environment (e.g., a healthcare facility, the health insurance system)—to regulate tension (stress) resulting from a situation (threat, loss, and challenge) that leads them to question their equilibrium (homeostasis); 2—the dynamics of the environment (ecosystem level), which includes the interrelationships over time between A/Os that self-regulate, as well as the more or less transient ecological niches (i.e., context, culture, and society) that permeate and condition the regulations.

The DEA-A framework, through the adaptation paradigm (homeostasis and allostasis) [17,18,20,25,76], proposes a trans-theoretical articulation between various recognized conceptual approaches in psychology, including stress and illness adjustment/coping models [34,36,38,62], behavioral change models [9,14], and ecosystem perspectives on human development [83,84], but also draws from various system/organization theories in Public Health research [e.g., [87,88]). The contribution of the DEA-A model does not lie in its paradigms, taken from these theories and validated in the literature, but in their articulation. To our knowledge, there is no framework that theorizes in the same model the complexity of both intra- and ecosystem functioning, which simultaneously describes the process of homeostasis/allostasis of the individual, extended to that of individuals in the entire ecosystem (e.g., psychology of actors) and that of organizations (i.e., organizational homeostasis), maps the adaptation of systems from the onto- to the macrosystem, including the chronosystem, and conceptualizes the notions of allostatic load and symbiotic regulation (i.e., which contributes to the proper functioning and synergy of the ecosystem). Such integration is, we believe, necessary to promote lasting changes in GH. Indeed, the framework could potentially assist in preventing six axioms that may limit our understanding of human or even organization functioning in the future, and, therefore, the scope of interventions in GH:

1. *The omnipotence of perception over the internal and external environment.* The DEA-A framework emphasizes perceptual appraisal but stresses that it serves as only a partial mediator. It should not overshadow either the objective impact that the environment has on resources (such as functional consequences of illness) or the role that resources play in the regulation process (such as having the necessary skills and opportunities, absent which, allostatic load may increase). Therefore, it prevents the fall into psychologizing the problem by neglecting the concrete resources and preexisting difficulties encountered by A/Os;
2. *The omnipotence of cognition over emotion*. The DEA-A framework emphasizes reflexive, but also automatic processes, as key features underpinning human functioning. It envisions the individual as in a constant struggle for self-regulation, with allostatic load undeniably affecting cognitive processes and consequently generating new emotional responses on a cyclical basis. In addition, it is possible that instinctive or automatic responses (e.g., stimulus–response conditioning, reflexes) may bypass cognition in some cases with a direct relationship between emotion and regulation. Applied to an organization, this may entail following a standard procedure or triggering automatic responses without considering the problem at hand;
3. *The omnipotence of self-determination over other motivations*. The DEA-A framework underlines that self-determination is indeed achievable, but it requires significant resources on the part of A/Os. When conditions permit, they can develop and exert control over their regulations. Alternatively, motivations remain autonomous, dependent on changes in the environment (including changes induced by allostasis). As a result, the A/O may have multiple conflicting motivations. Identifying and attributing such motivations may be influenced by cognitive (evaluation) biases;
4. *The omnipotence of behavior over other regulations*. In the DEA-A framework, emotion-oriented and cognition-oriented forms of regulation can be just as, or even more, appropriate than behavioral ones, depending on the situation that requires adaptation. Hence, such allostatic responses should be promoted in both individual-based and population-based interventions whenever appropriate. In addition, these alternative forms of regulation may also be equally or more costly than a behavior (for example, diagnosis acceptance or behavioral inhibition rather than action-taking in cases of aggressive behavior or obsessive–compulsive disorder);
5. *The omnipotence of intrasystem over ecosystem*. The DEA-A framework considers the environment as a living ecosystem rather than a passive one, requiring as much attention as for an A/O’s intrinsic functioning. The environment can bring both urgent adaptation demands and resources, as well as obstacles to these adaptations. In essence, allostasis, and by extension interventions or tools, are neither functional nor dysfunctional in themselves, but rather depend on their function in context, within a specific ecosystem;
6. *The omnipotence of mankind over nature*. The DEA-A framework refers to the homeostasis/allostasis process shared by other animal or plant species, which accounts for certain constants in the adaptation of organisms. Hence, it conveys the importance of not losing sight of the most parsimonious explanation, according to which human beings, and the organizational/institutional systems they erect, are concerned primarily and foremost with their own survival. Thus, the question of whether such adaptations are carried out in harmony with, or at the expense of, the other organisms in their ecosystem must be raised.

In keeping with a GH perspective, such epistemological considerations were deemed necessary to meet the challenges posed by the ecological and societal transitions of our time. The DEA-A framework aims to enable the establishment of a functional analysis, allowing us to understand why and how and diagnose with what consequences each system (living organisms, individuals/actors, organizations and institutions, geopolitical systems, etc.) adapts to and contributes to the situation. A diagnosis of this nature may result in recommendations that would promote more functional adaptations from stakeholders in symbiosis with their living ecosystem. In predicting future crises, the framework might be useful for identifying the constants of stability for each pivotal system. The purpose of this is to establish surveillance and secure these indicators over time (e.g., crisis observatory), thus avoiding possible domino effects.

Methodologically, complex theory-driven intervention-planning protocols such as Intervention Mapping [89,90] [IM] could be used to operationalize the model. An article containing guidelines and tools for applying the DEA-A framework through IM is presented elsewhere [91]. The approach involves the functional analysis of the ecosystem, the formulation of change objectives within the allostasis of A/Os, and the search for symbiosis. The use of an ecosystem process-of-change model, similar to the DEA-A, has already enabled the development of an intervention designed to support a sustainable return to work after breast cancer [92]. Researchers and GH providers who undertake to use the DEA-A model as a framework will opt for longitudinal designs or network analyses capable of capturing the adaptive trajectories of A/Os and their interactions over time [93], in particular the functionality (including allostatic load) of allostasis responses at both the intra- and ecosystem levels. The forms taken in the study context by the key features of the model (i.e., homeostasis constants, stress responses, allostasis, and ecosystem properties) will be identified for each A/O using mixed studies that allow for the contextualization of observations in real-life situations [94]. The use of complex AI-based models will be a preferred option in fundamental and experimental research based on the DEA-A model.

Although it articulates recognized and scientifically validated paradigms, the DEA-A model itself still remains to be validated empirically, e.g., through such AI models. Conceptual difficulties were, moreover, encountered, given the porosity of certain constructs or their scalability in the adaptation process. It was also not possible to be exhaustive in our illustrations given the very broad field of application of the DEA-A. Future contributions focused on case studies in GH still need to be carried out in order to specifically deepen, clarify, and illustrate the application of the DEA-A model in different contexts.

## 5. Conclusions

The DEA-A framework does not claim to establish, with illusory comprehensiveness, a full set of predictive links explaining how systems adapt to their dynamic environments. Therefore, it aims to be more flexible so that professionals may apply it to any field of GH research (e.g., ecological transitions, improving diagnosis and care pathways, clinical management, etc.) regardless of the experience and expertise involved (i.e., the universality of the framework facilitates its application to integrated care or research approaches, such as interdisciplinary and/or participatory approaches). The shift in epistemology requires us to rethink our methods and tools. A complementary contribution, therefore, provides practical guidelines and a suitable methodology for implementing the DEA-A framework [91].

## Figures and Tables

**Table 1:** Illustration of key components of the DEA-A model at intrasystem level.

DEA-A Key Components		Illustrative Constructs and Determinants from the Literature
Demands	Internal demands	Structural pressures to ensure basic system functions (nutrition, relationship, reproduction, maintaining the integrity of the organism); Illness physical consequences (organ-function depletion)
	External demands	Opportunity; Transitions/Life events (e.g., moving, death/loss); Situations of deafferentation/anomie; Hazard; Social pressure
Cognitions about issues		Primary Appraisal (Loss/Threat/Challenge); Attitude; Perception of risk in terms of severity, susceptibility, and imminence (temporal framing)
Resources	Internal/ Personal resources	Conditions (physical/mental health, professional status, social position); Psychological resources, i.e., Knowledge; Skills; Abilities; Intelligence (creative, analytical, practical); Executive Functions (e.g., planning, inhibition abilities, self-regulation); Flexibility (e.g., resource selection, optimization, and compensation capabilities); Interactional resources (Social Codes; Social Intelligence; Language/Communication, Empathy); Economic resources (e.g., properties, material goods, financial).
	External/ Ecosystem resources	Network of health professionals; Access to resources (e.g., access to care); Mobility; Social support (e.g., family/non-family caregivers, tutors, role models, service providers); Quality of relationships/interactions (e.g., therapeutic alliance, communication); Aid (e.g., subsidies); legislations; Equipment available; Time
Cognitions about control		Agency; Perceived Efficacy; Perceived Self-Efficacy; Evaluation of the temporality of the effects; Perceived risk of the solution
Stress		Physiological responses (e.g., hunger, thirst, fatigue, excitement); Sensations (e.g., numbness/ankylosing, nervous tension); Emotions (e.g., fear, sadness, disgust, anger, surprise, joy, boredom); Normative discomforts (e.g., cognitive dissonance, guilt, feeling of unfairness)
Allostatic regulation	Emotional	Desensitization; Breathing; Tolerance; Expression of emotions (e.g., crying); Apathetic withdrawal; Emotional Overload/Substitution
	Cognitive	Denial/Denial; Distortion (minimization/trivialization, exaggeration, e.g., of the threat/resource balance); Positive reassessment of the situation; Rationalization; Comparative/unrealistic optimism; Cognitive Avoidance/Distraction; Search for meaning/Causal attribution; Anticipation/Projection; Planning
	Behavioral *	Practice of an activity (e.g., physical, social, professional); Seeking Social Support; Acting out; Therapeutic compliance with recommendations; Problem management and prevention behavior; Socialization/Transformation/Arrangement of living space; Seeking of rehabilitation; Apprenticeships; Resource production; Reflexes (e.g., natural change of posture).
Allostatic load **		Decision-making tension; Ambivalence; Competitive Stress/Rebound tensions; Cognitive dissonance; Allostasis dysregulation: [immune] hyperactivation (overstress) or hypoactivation (understress); Cognitive overload; Psychological exhaustion (Burnout)
Feedback		Effects of Regulation on the internal/external environment; Reappraisal; Reinforcement/Punishment (positive or negative); Personal/Vicarious experience; Verbal Persuasion

Note: The table is indicative due to the porosity of the constructs and paradigms, but readers should be able to get a good idea of the contents associated with the components of the DEA-A model from it. * The activity is classified as BR because it modifies the external environment (e.g., by providing a means for meeting people) and the internal environment (e.g., by improving the physical condition of the person). Whether it is to alleviate tension, distract oneself, or organize one’s thoughts, it is the initiation of the behavior that has permitted the other ER and CR allostasis to occur. In the same way, the search for social support is a conduct aimed at increasing the capacity of support, therefore, providing resources for initiating/maintaining subsequent ERs, CRs, and/or BRs. ** In one respect, allostatic load overlaps with pressure and stress in that it determines the cost of adaptation.

## Data Availability

No new data were created or analyzed in this study. Data sharing is not applicable to this article.
